# 671. Introduction of simultaneous *Clostridioides difficile* Antigen and Toxin A and B detection for diagnosis of *C. difficile* related diarrhea at Michael E. DeBakey VA Medical Center

**DOI:** 10.1093/ofid/ofad500.733

**Published:** 2023-11-27

**Authors:** Patrycja Ashley, Alison Robins, Rosalba Gomez Morones, Claudia Aguayo-Millan, Daniela Morales-Aseff, Maria C Rodriguez-Barradas

**Affiliations:** Baylor College of Medicine, Houston, Texas; Baylor College of Medicine, Michael E. DeBakey VA Medical Center, Housotn, Texas; Baylor Collage of Medicine, Houston, Texas; Baylor College of Medicine, Michael E. DeBakey VA Medical Center, Housotn, Texas; Baylor College of Medicine, Michael E. DeBakey VA Medical Center, Housotn, Texas; Michael E. DeBakey VAMC and Baylor College of Medicine, Houston, Texas

## Abstract

**Background:**

Nucleic acid amplification test (NAAT) for diagnosis of *Clostridioides difficile* infection (CDI) is highly sensitive but is unable to distinguish colonization from infection. Antigen/toxin testing on the other hand may have lower sensitivity, especially early in infection with associated delay in diagnosis.

**Methods:**

We conducted a retrospective study at Michael E. DeBakey VA Medical Center prior and post the introduction of simultaneous *C.difficile* GDH Antigen and Toxin A/B (GDH/Tox) detection.

**Results:**

In the pre-intervention period 83 patients underwent NAAT, 16 patients had a positive test result (19.3%). In the post-intervention 120 patients were tested; 99 (82.5%) were negative for both GDH Antigen and toxin A/B (GDH-/tox-), 16 (13.3%) tested positive for the GDH Antigen but negative for Toxin A/B (GDH+/tox-) and 5 (4.2%) tested positive for both Antigen and Toxin (GDH+/tox+). Two patients (1.7%) who initially tested GDH+/tox- were subsequently retested due to persistent symptoms or evidence of colitis and were noted to have GDH+/tox+ result.

The rate of positivity of *C.difficile* testing in our institution decreased form 19.3% to 4.2% (p< .001) post introduction of the GDH/toxin A/B detection. The treatment rate of patients who had the test performed decreased from 18.1% to 9.2% (p=.062).

30-day readmission rate for any reason in the group that tested positive for CDI in the pre-intervention group was 18.5% (3 patients), with 30-day all-cause mortality 6.25% (1 patient). In the GDH+/tox+ and GDH+/tox- groups in the post-intervention period, the 30-day readmission rate for any cause was 9.5% (2 patients), and 30-day all-cause mortality was 6.25% (1 patient).

CDI testing results before and after the intervention.

**Figure:**
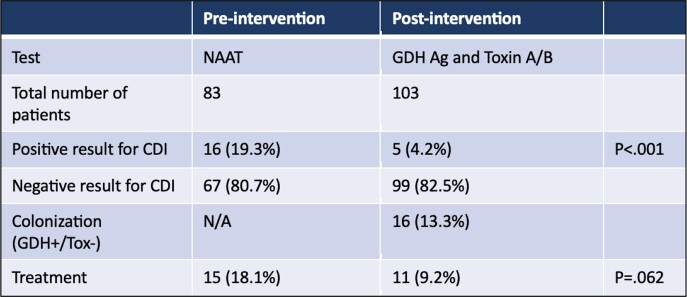

**Conclusion:**

Distinguishing asymptomatic carriage or colonization from CDI remains an important goal. Implementing GDH Antigen and toxin A/B *C.difficile* testing can help reduce the risk of inappropriate treatment of colonization and possibly reduce the risk for development of antibacterial resistant *C.difficile* strains, however in our case it initially missed 2 cases of CDI that fortunately was not associated with negative outcome. With use of the GDH/tox test repeat testing and/or treatment might be considered on case-by-case basis depending on persistence of symptoms or high suspicion of infection.

**Disclosures:**

**All Authors**: No reported disclosures

